# Off-Target Effect of Lovastatin Disrupts Dietary Lipid Uptake and Dissemination through Pro-Drug Inhibition of the Mesenteric Lymphatic Smooth Muscle Cell Contractile Apparatus

**DOI:** 10.3390/ijms222111756

**Published:** 2021-10-29

**Authors:** Matthew Stephens, Simon Roizes, Pierre-Yves von der Weid

**Affiliations:** Inflammation Research Network, Department of Physiology and Pharmacology, Cumming School of Medicine, University of Calgary, 3330 Hospital Drive N.W, Calgary, AB T2N 4N1, Canada; matthew.stephens@ucalgary.ca (M.S.); roizes@ucalgary.ca (S.R.)

**Keywords:** statins, lymphatics, smooth muscle, RhoKinase, cholesterol

## Abstract

Previously published, off-target effects of statins on skeletal smooth muscle function have linked structural characteristics within this drug class to myopathic effects. However, the effect of these drugs on lymphatic vascular smooth muscle cell function, and by proxy dietary cholesterol uptake, by the intestinal lymphatic network has not been investigated. Several of the most widely prescribed statins (Atorvastatin, Pravastatin, Lovastatin, and Simvastatin) were tested for their in-situ effects on smooth muscle contractility in rat mesenteric collecting lymphatic vessels. Lovastatin and Simvastatin had a concentration-dependent effect of initially increasing vessel contraction frequency before flatlining the vessel, a phenomenon which was found to be a lactone-ring dependent phenomenon and could be ameliorated through use of Lovastatin- or Simvastatin-hydroxyacid (HA). Simvastatin treatment further resulted in mitochondrial depolymerization within primary-isolated rat lymphatic smooth muscle cells (LMCs) while Lovastatin was found to be acting in a mitochondrial-independent manner, increasing the function of RhoKinase. Lovastatin’s effect on RhoKinase was investigated through pharmacological testing and in vitro analysis of increased MLC and MYPT1 phosphorylation within primary isolated LMCs. Finally, acute in vivo treatment of rats with Lovastatin, but not Lovastatin-HA, resulted in a significantly decreased dietary lipid absorption in vivo through induced disfunction of mesenteric lymph uptake and trafficking.

## 1. Introduction

Lymphatic vessels transport lymph, consisting of interstitial fluid, immune cells, antigens, and other macromolecules, to the lymph nodes for antigen screening and then into blood circulation [1]. The flow of lymph is perpetuated by an active process of lymphatic pumping, mediated by smooth muscle cells within the lymphatic vessel wall [2]. Lymphatic pumping is tightly regulated by intrinsic and extrinsic factors, many of them derived from the endothelial or smooth muscle cells [3,4]. These factors include chemical mediators such as nitric oxide (NO) or prostaglandins [5,6,7,8].

The lymphatic system plays a critical role in osmoregulation, immune cell trafficking, and adaptive immune response. While lymphatic disfunction is frequently observed at sites of inflammation and infection, it is commonly overlooked due to an incomplete understanding of its role in the disease’s pathology. These changes result in a variety of outcomes, including decreased fluid drainage (lymphedema) and reduced nutrient absorption (malnutrition). 

Statins are a group of successful cholesterol lowering medications. They are all competitive inhibitors of 3-hydroxy-methylglutarl-CoA (HMG-CoA) reductase, the rate limiting enzyme in the biosynthesis of cholesterol [9]. Therefore, statin-therapy potently reduces hypertension and the risk of cardiovascular diseases. Unfortunately, statin treatment sometimes results in patients developing myopathies, pain, and weakness in skeletal muscle, which in rare cases can lead to fatal rhabdomyolysis [10,11,12,13,14]. While, all commercially available statins have been reported to cause muscle-related side effects, the occurrence of such events is too inconsistent to identify the direct cause in the patient cohort [15]. Interestingly, the mechanisms underlying this toxicity have been partially identified, although not fully elucidated. Schirris and colleagues recently suggested an off-target interaction of lactone rings, structures found within some statins, were inhibiting mitochondrial respiration, thus inducing apoptosis, a phenomenon that was reduced with the use of the hydrolyzed statin forms [11]. It was also reported that in isolated muscle fibers, certain statins induced the release of Ca^2+^ from the sarcoplasmic reticulum thereby directly affecting the function of the ryanodine receptor RyR1—the predominant receptor involved in skeletal muscle calcium release. The experimental evidence was supported by naturally occurring mutations surrounding RyR1 within the patient cohort, which is associated with many serious musculoskeletal diseases [11].

Lipophilicity of statins has previously been correlated to increased occurrences of myopathies. Lipophilic drugs increase dissemination throughout the body as they have an increased ability to passively diffuse through thin membrane barriers and single cell barriers such as capillaries, endothelial layers, and the blood brain barrier. Importantly, the lymphatic system participates directly in the increased uptake of lipophilic molecules. This allows for medications, such as lipophilic-statins, to be given at lower dosages but maintain their biological impact [16].

Simvastatin and Lovastatin are two of the most prescribed statins and are available in a lipophilic lactone close-ringed form or an open-ringed hydroxyacid form. Both medications are regularly prescribed in their closed lactone form, which is converted in vivo, into their active form. With previous groups demonstrating the effect of statin prodrug-forms on skeletal muscle function, we hypothesized that a similar effect would be seen in vascular smooth muscle, specifically those of the lymphatic system. Here, we tested several statins for their acute effect on lymphatic vascular function. We describe novel mechanisms of actions of the closed lactone-ringed statins, Lovastatin and Simvastatin on mesenteric lymphatic vessel function through interference with key components of the lymphatic smooth muscle contractile apparatus. The resulting lymphatic pumping alteration has a biological effect on dietary lipid uptake and dissemination in vivo, suggesting a non-canonical means of reducing cholesterol levels through statin therapy independent of the targeted HMG-CoA-reductase inhibitory function. 

## 2. Results

### 2.1. Lovastatin and Simvastatin Treatment Result in Concentration-Dependent and Reversible Contractile Disfunction in Mesenteric Vessels

**Figure 1:**
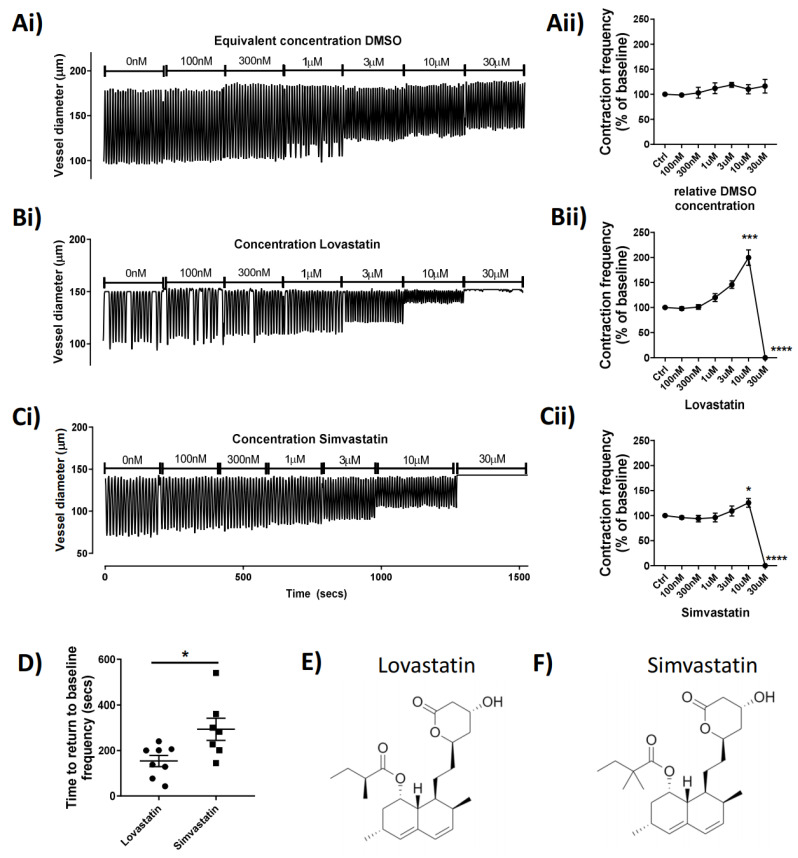
Concentration-dependent effect of statins on the contractibility of rat mesenteric collecting lymphatic vessels in situ. Isolated and pressurized rat mesenteric lymphatic vessels were incubated with increasing concentrations of the statins or a relative concentration of DMSO (**Ai**–**Ci**) Representative traces monitored for a 3 min period at each concentration. (**Aii**–**Cii**) Contraction frequency of vessel expressed as baseline-corrected percentage for each vessel tested. (**D**) Analyzed time for flatlined vessel to return to baseline contraction after statin treatment. Skeletal structures of (**E**) Lovastatin and (**F**) Simvastatin drawn using ChemDraw. Vessel contraction frequency is represented as mean ± SEM of *n* = 5–12 individual experiments. * *p* < 0.05, *** *p* < 0.001, **** *p* < 0.0001 vs. baseline. One-way ANOVA with Dunnet’s multiple comparisons test.

To investigate the potential acute effect of statins on lymphatic contractile function, we administered the statins via superfusion of isolated rat mesenteric lymphatic vessels mounted on a pressure myograph. The data show that Lovastatin (trade name Mevacor) and Simvastatin (Zocor) increased vessel contraction frequency in a concentration-dependent manner up to 30 µM, when they both completely abolished lymphatic contractility (*p* < 0.0001; Figure 1B,C). The example pressure myography traces (Figure 1Ai–Ci) show the concentration-dependent increase in vessel contraction frequency caused by Lovastatin and Simvastatin (reaching statistical significance at 10 µM) (Figure 1Bii,Cii). Vessels then quickly flatlined within 5–10 s after addition of Lovastatin or Simvastatin at 30 µM following a burst of activity. Interestingly, at all concentrations tested, neither Pravastatin nor Atorvastatin demonstrated any significant effect on pumping compared to DMSO controls (Supplemental Figure S1). Furthermore, vessel activity could be restored completely upon washout of Lovastatin and Simvastatin; however, the vessel recovered significantly more quickly from treatment with Lovastatin (median 169 s) than with Simvastatin (median 282 s) (*p* = 0.0155) (Figure 1D). Derived lactone metabolites isolated from Aspergillus terreus, the chemical structures of the pro-drug forms of Lovastatin [17] and Simvastatin [18], are almost identical in composition, differing only by a single methyl-group (Figure 1E,F).

### 2.2. Lovastatin and Simvastatin’s Functional Effect on Lymphatic Vessel Contractility Is Lactone Ring Dependent

**Figure 2:**
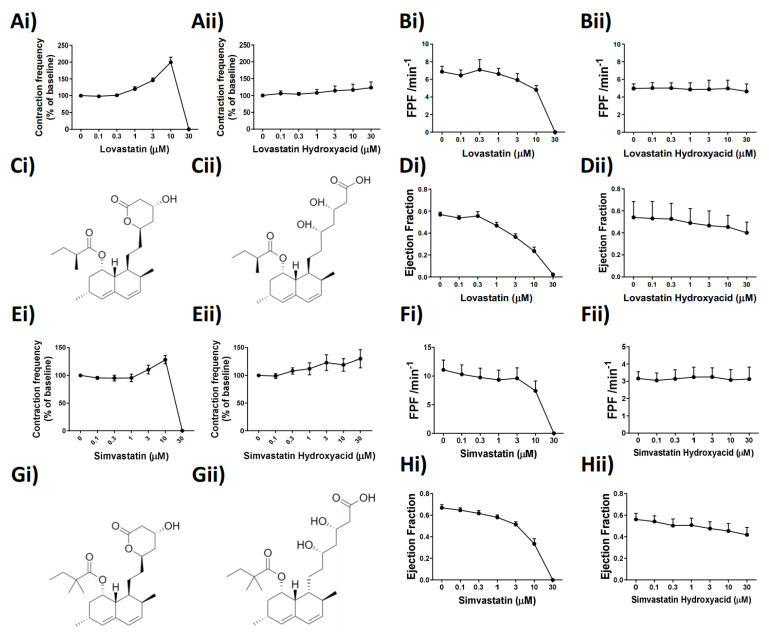
The lactone ring structures of Lovastatin and Simvastatin are intrinsic to the statin-induced alteration of mesenteric lymphatic vessel contraction frequency, ejection fraction, and fractional pump flow. (**Ai**) Lovastatin vs. (**Aii**) Lovastatin-hydroxyacid (contraction frequency/baseline (%) concentration–response curve) (**B**) Calculated fractional pump flow (FPF) and (**D**) ejection fraction (EF) of vessels in response to concentration-dependent stimulation of statin used in (**A**) and (**E**), respectively. (**Ei**) Simvastatin vs. (**Eii**) Simvastatin-hydroxyacid (contraction frequency/baseline (%) concentration–response curve). (**Fi**,**Fii**) Calculated Fractional pump flow (FPF) and (**Hi**,**Hii**) ejection fraction (EF) of vessels in response to a concentration-dependent increase in Simvastatin or Simvastatin Hydroxyacid. (**C**,**G**) Skeletal chemical structures of drugs used in panels i and ii. *n* = 4–12 lymphatic vessels from 4–12 different rats. Chemical structures drawn using ChemDraw.

While the precise effect of Lovastatin and Simvastatin on the lymphatic vessel function was unknown, we examined whether it could be, in part, structurally based. Identification of key structural characteristics, conserved between the statins tested, revealed the presence of a lactone ring structure in only Lovastatin and Simvastatin, for which we used the typically prescribed pro-drug (Figure 2Ci,Gi). We thus hypothesized that the presence of the closed lactone ring structure in the unprocessed pro-drugs was the reason for their effects on lymphatic contractility and that we may be able to alleviate those through use of the hydrolyzed (open lactone ring), active drug forms (Figure 2Cii,Gii). To test this hypothesis, pressurized, isolated rat mesenteric collecting lymphatic vessels were subjected to a concentration-dependent stimulation of Lovastatin, Simvastatin, or their hydroxyacid forms. Paired vessel analysis demonstrated that at equal concentrations the vessel pumping inhibitory effects of Lovastatin (Figure 2Ai) and Simvastatin (Figure 2Ei) were not observed with the respective hydroxyacid forms (Figure 2Aii,Eii), supporting the role of the lactone ring driving its functional effect. The loss of fractional pump flow (FPF) (Figure 2Bi,Fi) and ejection fraction (EF) (Figure 2Di,Hi) witnessed with the concentration response curve to the pro-drug forms of Lovastatin and Simvastatin were completely absent with the use of the hydroxyacid derivatives (Figure 2B,D,F,Hii).

### 2.3. Simvastatin but Not Lovastatin Causes Acute Mitochondrial Fission and Actin Depolymerization in Lymphatic Smooth Muscle Cells Unrelated to Acute Functional Effects

**Figure 3:**
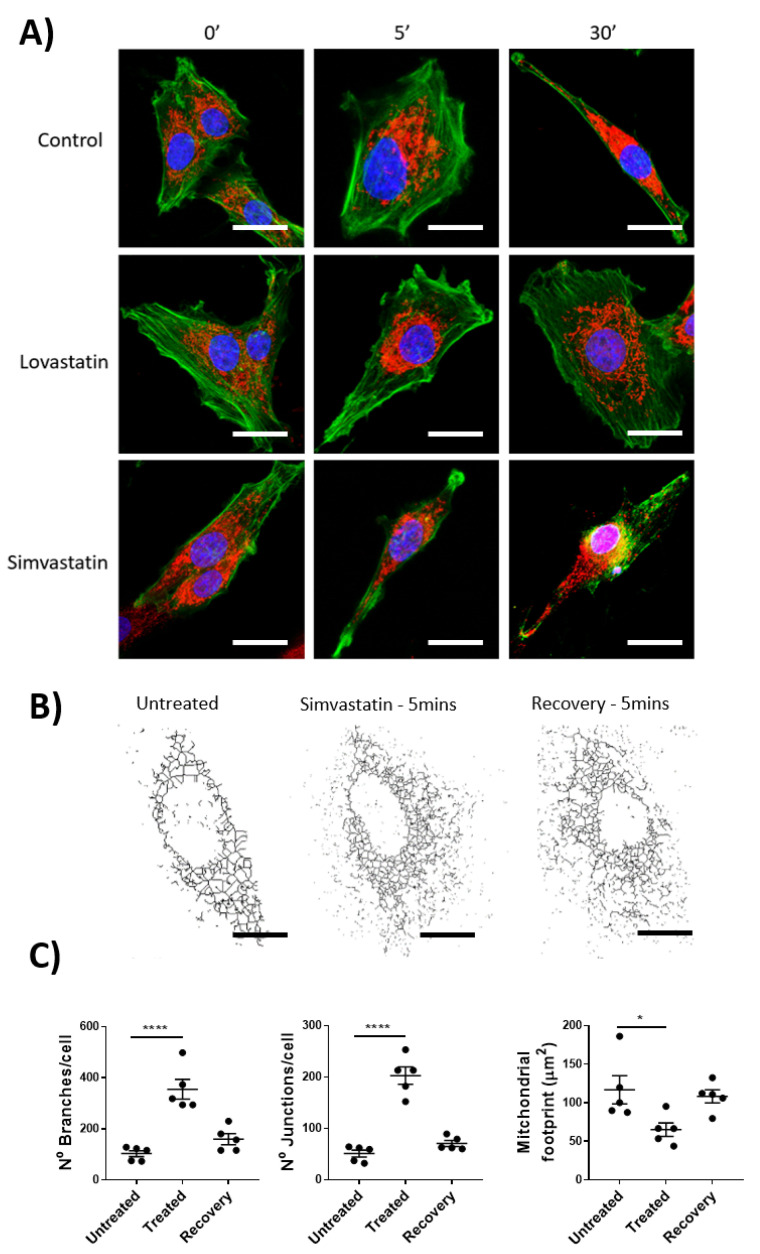
Mitochondrial network fission in mesenteric lymphatic smooth muscle cells of Simvastatin-treated rats. Primary LMCs were treated in vitro with 10 µM of Lovastatin or Simvastatin for 5 or 30 min before being fixed and stained for the condition of the mitochondrial and actin cytoskeletal network using MitoTracker and Phalloidin, respectively. (**A**) Representative confocal images of individual cells and the effect of 5 or 30 min of treatment with each statin or control (Green: Phalloidin, Red: MitroTracker Red CMXRos). (**B**) Mitochondrial network structural analysis of a representative effected cell treated with Simvastatin for 5 min (Treated) or wash-out for 30 min (Recovery). (**C**) Images were analyzed for Mitochondrial Branches, junctions, and overall mitochondrial footprint. Scale bar = 10 µm. Data are presented as the average ± SEM of 5 randomly picked cells from *n* = 3 experiments. * *p* < 0.05, **** *p* < 0.0001 vs. untreated cells One-way ANOVA with Dunnet’s Multiple comparison test.

With previously published data highlighting the inhibitory effects on the off-target mitochondrial complex III by lactone-ringed statins (including Lovastatin and Simvastatin) and their association with skeletal muscle myopathies, we hoped to evaluate whether this effect was seen within lymphatic smooth muscle. Specifically, isolated rat mesenteric lymphatic smooth muscle cells were incubated with Lovastatin or Simvastatin and their mitochondrial health measured by mitochondrial network assessment (via MitoTracker CMXROS staining) and F-actin distribution (via Phalloidin counterstaining of fixed cells). As the lymphatic vascular effect of Lovastatin and Simvastatin was noted within a short period of time (<30 s), we wanted to investigate whether the mitochondrial network was disrupted by comparatively short (5 min) or long (30 min) exposure. The data shows that Lovastatin treatment had no significant effect on gross cell morphology, mitochondrial network dynamics, or F-actin distribution (Figure 3A,C), whereas 5 or 30 min of treatment with Simvastatin caused significant cell changes as indicated by actin depolymerization and mitochondrial fission (Figure 3A). Further analysis of the mitochondrial network using ImageJ mitochondrial network analysis, as previously described [19], indicated an increased number of branches and junction per cell and a decreased mitochondrial network surface area (Figure 3A–C). It is noteworthy that this mitochondrial effect could be resolved upon removal of Simvastatin and a 5 min recovery phase (Figure 3C). 

### 2.4. Lovastatin’s Acute Effect on Lymphatic Contractility Is Independent of Nitric Oxide or Prostaglandin Synthesis

**Figure 4:**
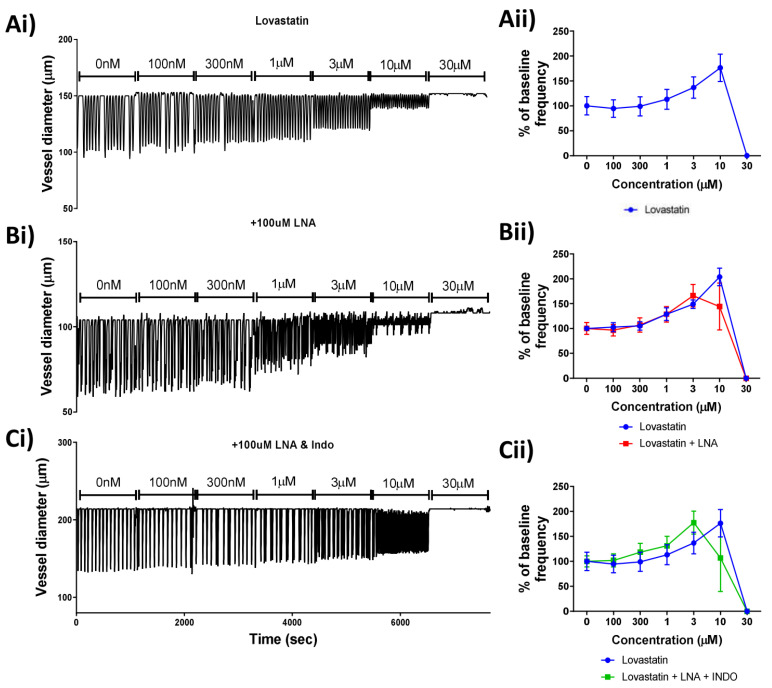
Effect of Lovastatin on the contractibility of mesenteric collecting lymphatic vessels is independent of nitric oxide and/or prostaglandin synthesis. Representative traces of isolated and pressurized rat mesenteric lymphatic vessels were incubated with increasing concentrations of (**Ai**) Lovastatin, (**Bi**) LNA pre-treated vessels, or (**Ci**) vessels pre-treated with LNA + Indomethacin. (**Aii**–**Cii**) Plots for vessel contraction frequency displayed as a percentage of baseline, monitored for a 5 min period at each incremental concentration of Lovastatin. Raw representative traces are shown, clipped to 5 min periods. Data are presented as the percentage of baseline frequency of individual vessels. *n* = 5–10 individual experiments.

Due to its lack of a mitochondrial-driven effect on the lymphatic smooth muscle cells, Lovastatin alone was investigated further. In order to determine whether nitric oxide (NO) or prostaglandin production was involved in the Lovastatin-induced lymphatic vessel contractility effect (Figure 4Ai), vessels were pre-treated for 20 min with L-NA (to block all nitric oxide synthase (NOS) isoforms) (Figure 4Bi) or a combination of L-NA and Indomethacin (inhibiting NOS and cyclooxygenases, COX) (Figure 4Ci) before challenge with a cumulative increase in Lovastatin concentration. Results indicated that neither NOS inhibition by L-NA nor COX inhibition via Indomethacin mitigated the response to Lovastatin (Figure 4Aii–Cii), suggesting the effect of Lovastatin on lymphatic vessel contraction is independent of NO or prostaglandin synthesis. 

### 2.5. Lovastatin Alters Mesenteric Lymphatic Contraction Frequency through Modulation of Rhokinase

**Figure 5:**
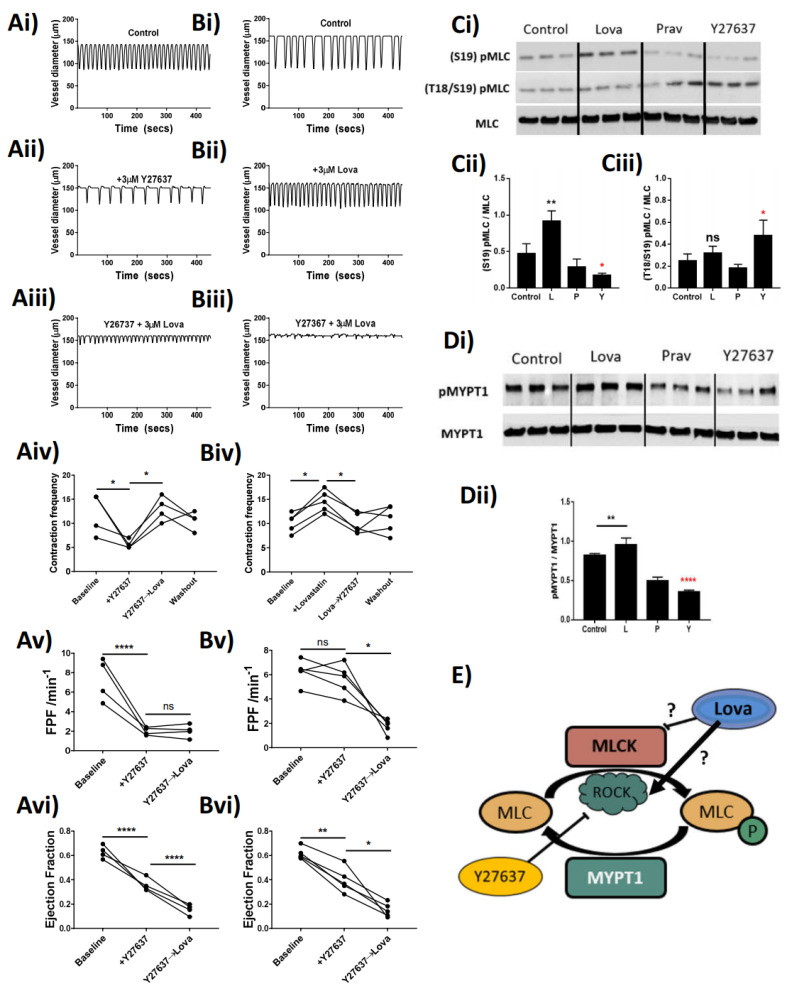
Lovastatin modulates the effect of RhoKinase inhibitor Y27637 in rat mesenteric collecting lymphatic vessels and isolated smooth muscle cells. Isolated and pressurized rat mesenteric lymphatic vessels were (**Ai**,**Bi**) baseline control, pre-incubated with either (**Aii**) 3 µM Y27637 or (**Bii**) 3 µM Lovastatin for 20 min before being co-incubated with (**Aiii**) 3 µM Lovastatin or (**Biii**) 3 µM Y27637. (**Aiv**,**Biv**) Vessel contractility frequency changes in response to treatments were assessed over a 5 min period after the addition of each mentioned drug. (**Av**,**Avi**,**Bv**,**Bvi**) Changes in Fractional pump flow (FPF) or Ejection fraction of individual vessels after addition of each drug. Primary isolated rat collecting lymphatic smooth muscle cells were incubated with DMSO control, Lovastatin (10 µM), Pravastatin (10 µM), or Y27637 (3 µM) and effects on the phosphorylation of (**C**) MLC2 and (**D**) MYPT1 were determined by Western blot and quantified in comparison to total protein expression. (**E**) Proposed mechanism of interaction whereby Lovastatin inhibits MLCK function, thereby promoting MLC2 phosphorylation and increased contractile frequency in mesenteric collecting lymphatic vessels. Pressure myography data are represented as the percentage of baseline frequency of individual matched vessels, *n* = 4 individual experiments. Western blots are representative blots of 3 independent replicates loaded side-by-side. Graphs are plotted as mean ± SEM. Statistical significance was determined using a single ANOVA of repeated measure with Sidak post-hoc test. * *p* < 0.05, ** *p* < 0.01, **** *p* < 0.0001, ns-not significant.

Through pharmacological inhibition of ROCK, with the small-molecule inhibitor Y27637, we attempted to dissect the mechanisms whereby Lovastatin alters contractile function. The data show that treatment of the vessels with 3 µM Y27637 resulted in a significant decrease in vessel contraction frequency and amplitude, which in turn caused a significant loss in FPF and EF (Figure 5Av,Avi). These effects could be rescued by additive treatment with Lovastatin, which returned the contractile frequency to baseline (Figure 5Aiv). However, the increased contraction frequency did not compensate for the loss of FPF and, in fact, significantly decreased EF of the vessel (Figure 5Avi). Reversed-order treatment with Lovastatin followed by Y26737 resulted in an initial increase in vessel contraction frequency, which was returned to baseline with ROCK inhibition (Figure 5Biv). The Lovastatin-induced increase of contraction frequency (Figure 5Biv) left the FPF unchanged (Figure 5Bv) by compensating for the significant loss of EF (Figure 5Bvi). This increased contraction rate could be restored back to baseline with Y27636 treatment culminating in a significant loss of both FPF and EF.

Data in Figure 5A,B highlight the ability of Lovastatin to directly impact the contraction frequency of the lymphatic vessel through interference with regulatory enzymes involved in the phosphorylation of MLC (ROCK) in a pressure myograph-based ex vivo assay. In order to further clarify Lovastatin’s direct impact on ROCK, we analyzed the contractile protein apparatus in primary cultured rat lymphatic smooth muscle cells. In smooth muscle cells, MLC2 is reported to be phosphorylated at Thr18 and Ser19 by MLCK in a Ca^2+^/calmodulin-dependent manner correlating with smooth muscle cell contraction [20]. ROCK, meanwhile, can also phosphorylate MLC2 but only at Ser19, which regulates the assembly of stress fibers [21]. Through the use of these primary cultured rat lymphatic smooth muscle cells, we demonstrated that Lovastatin could increase the basal phosphorylation state of Ser19-MLC2, while subsequently increasing phosphorylation at Thr18-Ser19 through treatment with Y27637. Notably, treatment with Lovastatin (but not Pravastatin, used here as an open lactone ring control) increased total Ser19-MLC2 phosphorylation but had no effect on Thr18/Ser19-MLC2, suggesting a Ca^2+^/calmodulin-independent action (Figure 5C). Assessment of the activity of the MYPT1 also demonstrated that Lovastatin significantly increased total phosphorylation of MYPT1, suggesting it was acting in a similar fashion as was ROCK in these cells (Figure 5D). We herein propose a novel mechanism (Figure 5E) by which Lovastatin either improves residual ROCK function or inhibits the function of MLCK in smooth muscle cells, thereby regulating lymphatic smooth muscle contraction in a calcium-independent manner. 

### 2.6. Acute Treatment of Lovastatin Reduces Dietary Lipid Absorption and Peripheral Dissemination In Vivo

**Figure 6:**
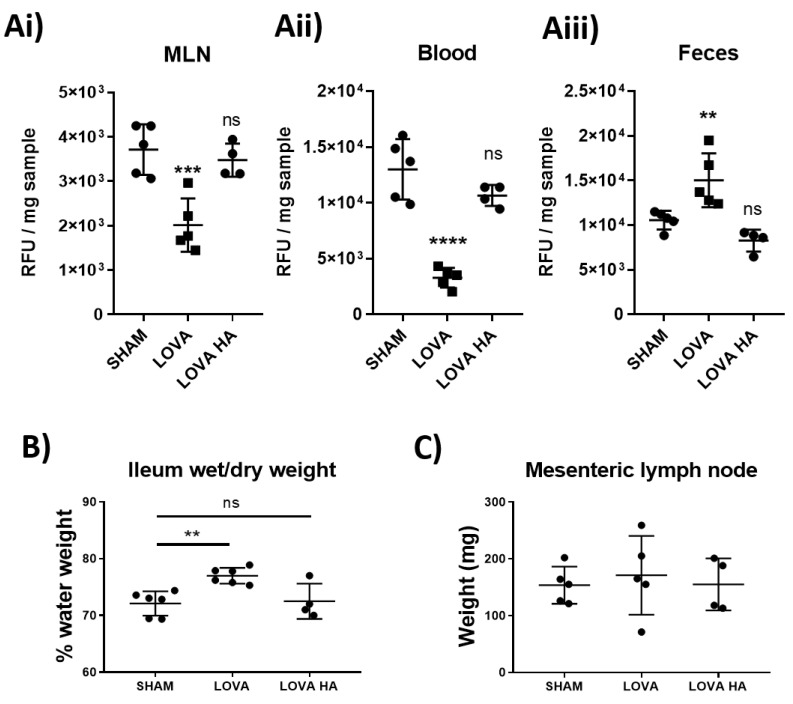
Pro-drug form of Lovastatin inhibits dietary lipid uptake in vivo. Rats were fasted for 4 h before treatment with a 10 mg/kg I.P. injection of either saline control, Lovastatin, or Lovastatin-hydroxyacid (HA) 1 h prior to being gavaged with a bolus of Bodipy-FL16 combined with oleic acid. 1 h after the bolus was given, (**Ai**) mesenteric lymph nodes (MLN), (**Aii**) blood, and (**Aiii**) feces were collected and analyzed for Bodipy content (see Methods). Additionally, fluid retention within the (**B**) ileum (ILE) and (**C**) MLNs were determined by wet/dry weight analysis. Data are presented as mean ± SEM of 5 rats over 5 independent experiments. ** *p* < 0.01, *** *p* < 0.001, **** *p* < 0.0001 vs. sham One-way ANOVA with Dunnet’s multiple comparison test. ns-not significant.

Statins drastically reduce cholesterol biosynthesis and circulating levels of cholesterol. We investigated whether the impact of Lovastatin on reducing lymphatic contractility could be an important contributing factor in the statins’ cholesterol lowering properties. We thus examined the effect of Lovastatin on the absorption and distribution of the fluorescent cholesterol-based compound Bodipy-FL16 administered to rats by gavage. The short-term effect of the administration of a single dose of Lovastatin (10 mg/kg) significantly impeded intestinal dietary lipid absorption, as indicated by reduced lipid accumulation in the mesenteric lymph nodes (Figure 5Ai), decreased serum fluorescent cholesterol levels (Figure 6Aii), and an accumulation of dietary lipids within the feces (Figure 6Aiii). These findings suggest a novel acute effect of Lovastatin treatment in vivo on its ability to decrease intestinal lipid absorption. This reduction in lipid absorption through decreased lymphatic contractility resulted in a mild, yet significant, edema within the ileum of the treated rats (Figure 6B) while not affecting the draining mesenteric lymph node weight (Figure 6C). Furthermore, we demonstrate that all these effects are specific to the use of the closed lactone-ring form of Lovastatin as, Lovastatin-hydroxyacid did not lead to significant alteration in cholesterol bio-distribution compared to sham controls, suggesting the conserved inhibitory effect of the lactone ring in vivo (Figure 6A–C).

## 3. Discussion

Statin-induced myopathies present a medical phenomenon with pathological implications. While prodrug forms of statins containing a lactone ring have been reported to bind to the Q_o_ site of mitochondrial complex III, providing an explanation for the association of some statins with skeletal muscle myopathies [11], it is unknown whether the effect is conserved in other systems containing smooth muscle. We hypothesized that lymphatic smooth muscle cells could be similarity affected, thereby altering their functional activity. 

In the present study, we aimed to elucidate the effect of popular commercialized statins on lymphatic vascular function, and we discovered a previously unreported effect of Lovastatin and Simvastatin on the vascular lymphatic system. We demonstrated that these statins in their pro-drug forms impair in a concentration-dependent manner the phasic and rhymical contractions of mesenteric lymphatic vessels, an effect that culminates with a complete loss of tone, suggesting an inability of the vessels to effectively propel lymph. Furthermore, the action of these two statins, while acute, is reversible and seemingly solely dependent on the lactone ring structures found within the pro-drug forms of both Lovastatin and Simvastatin (Figure 2). Other tested commercial statins (Atorvastatin and Pravastatin) that lack the lactone ring had no impact on lymphatic vessel function, suggesting the importance of this structural moiety (Supplemental Figure S1). 

The effects of statins on mitochondrial function is a developing field of research highlighting not only the deleterious effects of statin use, but also novel protective effects. As HMG-CoA is a key enzyme in the mevalonate pathways, its inhibition (by statins) results in the reduced availability of farnesyl pyrophosphate, geranylgeranyl pyrophosphate, heme A, coenzyme Q10, and other metabolites essential for cellular function [22]. Moreover, as cholesterol is not the end-product of these pathways, there can be a variety of pleiotropic effects with chronic use [23]. Lactone ring functional groups, contained within several lipophilic statins, have been recently presented as potential inhibitors of the Q_o_ of the mitochondrial cytochrome complex III subunit [11]. Using isolated rat lymphatic smooth muscle cells, paired with mitochondrial staining and imaging, we demonstrated that the pro-drug form of Simvastatin, but not Lovastatin, had a significant impact on mitochondrial dynamics. These data, while demonstrating a partially conserved mechanism of action between skeletal and vascular smooth muscle cells, highlight the ambiguity between cells of differing tissue origin, their associated function, and the impact of drug effects. We acknowledge that due to the absence mitochondrial-function assessment through specialized assays such as Seahorse^TM^, we cannot definitively prove the absence of a mitochondrial complex III inhibitory effect as has been previously published [11].

The collecting lymphatic vessel is comprised of an endothelial tube surrounded by specialized lymphatic smooth muscles. While the force and rhythmicity of lymphatic contractions is generated in the lymphatic smooth muscle layer, there is clearly documented regulation of pumping through molecules derived from the endothelium, specifically NO and prostaglandins [5,7,8]. Several reports highlighted an increased production of NO by statins in a way that is independent of its canonical HMG-CoA reductase inhibitory activity by either involving scavenger receptor-B1 [24] or LOX-1, a receptor for oxidized low-density lipoprotein [25]. Furthermore, statin treatment was also shown to induce the production of prostaglandins in rat liver [26]. As the production of either product would likely result in the reduction/ablation of vessel contractility that we observed, we performed a combination of ex vivo lymphatic vessel pressure myography and pharmacological interventions to assess whether the endothelium could play a role in the statin-induced lymphatic disfunction. Our findings determined that the action of Lovastatin on vessel contraction was independent of the production of either of these mediators. The highly specific role of FOXC2-positive lymphatic endothelial cells (such as those used here) for their controlled specialization and function within the gastrointestinal tract may provide an explanation for the differing findings when compared to the cardiovascular literature [27].

The phasic nature of lymphatic vessel contractility is intrinsically generated in lymphatic smooth muscle cells. As in other smooth muscles, this contraction–relaxation cycle relies on the phosphorylation–dephosphorylation of myosin light chains (MLC) regulating its impact on the actin cytoskeleton [21] and providing the necessary force for the propulsion of lymph. The regulation of MLC phosphorylation depends on the essential activity of regulatory kinases such as ROCK, myosin light chain kinase (MLCK), and myosin light chain phosphatase (MLCP) via subunits such as MYPT1. Statins act to inhibit the function of HMG-CoA reductase, and as such can have an indirect effect on the activity of ROCK through downregulation of the production of geranylgeranyl pyrophosphate and the associated rhoA synthesis [28]. There is also evidence within the literature that shows direct in vivo inhibition of ROCK itself by statins [29]. Our data demonstrate that Lovastatin treatment negates the effects of a ROCK inhibitor (Y27637) on lymphatic contractility, through a proposed action of increasing the function of residual ROCK within the system. We support this claim through the analysis of isolated primary cultured rat mesenteric lymphatic smooth muscle cells, whereby treatment with Lovastatin causes a hyper-phosphorylation of both MLC2 and MYPT1, proteins whose phosphorylation sites are regulated by ROCK. We propose that through this increased functionality of ROCK, the vessel stops contracting as the dephosphorylation of MLC is outweighed by active phosphorylation within the cell (proposed schematic Figure 5E). Our findings contrast published reports in which other statins (Atorvastatin) have been described to potently reduce ROCK activity in patients with atherosclerosis [29]. However, whether this is a direct inhibition of the functionality of ROCK or an indirect inhibition through another mechanism is still not fully elucidated and as such remains an area for further investigation. Furthermore, as that study assessed the levels of ROCK activity in circulating leukocytes, it is possible the effect, and therefore function of statins therapy, is unique between stromal and immune cells.

Analysis of lymphatic vessel function using pressure myography clearly demonstrated that Lovastatin treatment caused a significant loss in lymphatic vessel ejection fraction and fractional pump flow (Figure 5). We posited that this alteration would drastically reduce the ability of the intestinal mesenteric lymphatic system to disseminate dietary absorbed lipids. In line with this hypothesis, we tested and report on a pronounced disruption in dietary cholesterol uptake and dissemination in rats treated acutely with Lovastatin (Figure 6). This effect was confirmed to be prodrug specific as no impact was observed with treatment of Lovastatin hydroxyacid. These findings reveal a novel mechanism whereby statins actively lower circulating cholesterol by decreasing intestinal lymphatic lipid absorption, transport, and peripheral dissemination. The mechanism is promoted by the action of the lactone ring statin pro-form of Lovastatin, likely transported through the mesenteric lymphatic vessels prior to their enzymatic conversion during first-pass metabolism. 

Overall, our study highlights the off-target effect of Lovastatin and Simvastatin pro-form drugs on the lymphatic vasculature. Our novel findings outline a non-canonical mechanism by which Lovastatin regulates the function of the lymphatic smooth muscle cell contractile apparatus, an action that explains the decrease in cholesterol distribution we observed in vivo (Figure 5). While these data support our overarching hypothesis, we have outstanding questions. Currently we do not fully understand why the treated lymphatic vessels stop pumping in a dilated rather than contracted state. We propose that this dilation is caused by dissociation of pMLC2 from the actin cytoskeleton in a “cutting-of-strings” event that has yet to be witnessed and requires future investigation. 

## 4. Materials and Methods

### 4.1. Animals

Male Sprague Dawley rats (200–300 g, Charles River, Montreal, QC, Canada) were used to perform all experiments. Animals were housed at a room temperature of 22 °C with 12 h day-and-night cycles and were allowed full access to water and food at all times; both normal circadian rhythms and body temperatures were maintained. All animal protocols were reviewed and approved by the University of Calgary Animal Care and Ethics Committee and were conducted in accordance with the guidelines of the Canadian Council on Animal Care and the National Institutes of Health’s Guide for the Care and Use of Laboratory Animals (Certification No. AC16-187 and AC20-0140). 

### 4.2. Lymphatic Vessels Preparation

Rats were anaesthetized by isofluorane (Pharmaceutical Partners of Canada, Richmond Hill, ON, Canada) inhalation in a sealed box and euthanized by decapitation. The small intestine including jejunum and ileum was exteriorized after laparotomy and kept in albumin physiological saline solution (APSS; in mM: 145.0 NaCl, 4.7 KCl, 2 CaCl_2_, 1.2 MgSO_4_, 1.2 NaH_2_PO_4_, 5.0 Dextrose, 2.0 Sodium Pyruvate, 0.02 EDTA, 3.0 MOPS, and 5 g/L bovine serum albumin (BSA)) with pH adjusted to 7.4 at room temperature. Pieces of ileal mesentery containing mesenteric arcades (artery, vein, and two collecting lymphatic vessels on each side of the blood vessels) were isolated from the gut and loosely pinned down on a Sylgard-coated Petri dish filled with APSS until ready to use. Individual arcades were transferred to a clean, APSS-filled, Sylgard-coated Petri dish, set on the stage of a dissection microscope and collecting lymphatic vessels were then carefully dissected out of the mesentery and surrounding adipose tissue. 

### 4.3. Pressure Myography

Following dissection, vessels were placed in a 2 mL pressure myograph chamber (Culture Myograph, DMT-USA Inc., Ann Arbor, MI) filled with APSS with pH adjusted to 7.4 at 37 °C, and they were cannulated with two pulled and matched glass micropipettes (original size: OD 1.2 mm, ID 0.69 mm; approximate final ID 80–120 μm). Lymphatic vessels were tied at each end with a fine suture to the glass pipettes, which restricted fluid leakage as well as pressure drops inside the lymphatic vessel. Each pipette was connected to the same adjustable pressure reservoir filled with APSS (pH adjusted to 7.4 at 37 °C). The vessel containing chamber was then placed onto a stage with built-in heating over an integrated DMT inverted microscope. Having both pipettes connected to the same reservoir ensured that no flow was generated by pressure differences and pressure inside the vessel (transmural pressure) was constant and equal to that determined by the height of the single reservoir. The organ bath was superfused at a rate of 0.25 mL/min with APSS. Vessel leak, integrity, and twist were assessed first by applying a positive transmural pressure (3–5 cmH_2_O) and then approximate in situ length was established by briefly increasing the transmural pressure to 10 cmH_2_O. The vessel was then set to a transmural pressure of 3 cmH_2_O for a 30 min equilibration period, and then to 5 cmH_2_O for the duration of the experiments. Vessel contractions were recorded via a USB CCD video camera with the MyoView software [30]. To assess endothelial integrity, acetylcholine was added to the bath at a concentration of 10 μM and a transient (20 to 60 s) relaxation was observed. When used, blockers of eNOS, COX, and guanylate cyclase (N^G^-nitro-L-arginine (L-NA) or indomethacin, respectively) were added directly to the superfusion, vessels were then left for 20 min before applying further treatments. Endothelium-dependent responses were abolished in the presence of LNA or indomethacin. Statins were added directly into the bath and mixed by pipetting while superfusion was stopped during those applications to prevent dilutions. The rho-associated protein kinase (ROCK) inhibitor Y-27632 or myosin light-chain kinase (MLCK) inhibitor ML9 was added directly to the bath, and their effect on contractile activity occurred within minutes.

### 4.4. Western Blot

Isolated lymphatic vessels were lysed in extraction buffer, loaded on SDS polyacrylamide gels, and subjected to electrophoresis (120 V, 90 min). Proteins in the separating gel were then transferred to 0.2 μm nitrocellulose membranes (Bio-Rad Laboratories (Canada) Ltd., Mississauga, ON, Canada) and fixed by complete dehydration. A blocking solution of 5% BSA in 0.1% TBST (a mixture of Tris-buffered saline (TBS, 150 mM NaCl, 25 mM Tris HCl with pH 7.5) and Tween 20 (0.1%) with 5% bovine serum albumin (BSA) for 60 mins was employed to minimize non-specific binding. The membranes were then incubated with primary antibody targeting p-MLC (Ser18/19 and Ser20), MLC, pMYPT1, MYPT1 (all 1:1000 dilution; New England Biolabs Canada, Whitby, ON, Canada), and β-actin (1:10,000 dilution; Biolegend, San Diego, CA, USA) in blocking solution overnight, at 4 °C. After washing, the membranes were then incubated with the appropriate secondary antibodies, anti-Donkey or anti-Mouse IgG-horseradish peroxidase-conjugated secondary antibody (1:5000 dilution; Jackson Immunoresearch, West Grove, PA, USA), for 60 min. After extensive washing in TBST, the chemiluminescence signal was detected with West Dura Substrate (ThermoFisher Scientific, Burlington, ON, Canada). Images were acquired with the Bio-Rad Touch chemiluminescence imaging software and blots quantified using the open-source image processing package Fiji (Previously ImageJ) [31,32]. Levels of phosphorylated MLC or MYPT1 were normalized to their total protein expressions, respectively.

### 4.5. Assessment of Mitochondrial Function via Immunofluorescence Imaging

Lymphatic smooth muscle cells were kindly provided by Dr Sanjukta Chakraborty (Texas A&M University) and were cultured as published [33]. Cells were plated on 8-well chamber slides 48 h prior to stimulation at 2000 cells/well. Before treatments, cells were pulse-chased with Mitotracker, CFMXrROS (Thermo Fisher Scientific, Burlington, ON, Canada) for 30 min. After treatments, cells were immediately fixed in cold 4% formalin for 20 min without agitation and then transferred to PBS. Cells were stained with Alexa-488 conjugated Phalloidin for 20 min (1 ng/mL; Thermo Fisher Scientific, Burlington, ON, Canada) in PBST before slides were washed and mounted in Vectorshield Fluosave with DAPI (Vector Laboratories, Burlingame, CA, USA). Slides were imaged immediately on a Leica SP8 inverted confocal microscope and images were post-processed using Leica’s LASX software [34]. Images were taken using sequential scanning on three separate channels to remove all possible overlap near the laser and all images were taken at 63× oil immersion at designated digital zoom. Five cells per treatment were randomly selected, imaged, and the mitochondrial network was assessed as previously published.

### 4.6. Lymphatic Function Assessment

After a two-hour fasting period, rats were injected intra-peritoneally with 5 mg/kg of the statin of choice. Two hours after statin injection, animals were gavaged with 300 µL of oelic acid containing 100 µg of Bodipy.FL C16 (Thermo Fisher Scientific, Burlington, ON, Canada) One-hour post-gavage, rats were euthanized as described above. The mesenteric lymph node, fecal pellet, and blood were collected for downstream processing. Blood was left to coagulate at room temperature for 15 min before being spun at 3000× *g* for 10 min to clarify the serum that was then collected and kept on ice. Mesenteric lymph nodes and fecal pellets were weighed and 10× *w*/*v* of sterile PBS was added to the sample. Each sample was then homogenized through a 100 µm filter to remove large debris and cells separated by centrifugation at 3000× *g* for 10 min. The clarified supernatant was collected and kept on ice. Total Bodipy concentration was then analyzed using a Victor X3 fluorescent plate reader (FITC ex 488 nm/em 535 nm) and compared to solution and sample auto fluorescent values from non-gavaged rat controls. All values were then baselined against the reading in wells that only contained PBS.

### 4.7. Chemicals

Statins were purchased from Cayman Chemical (Ann Arbor, MI, USA) and all other chemicals were from Millipore Sigma (Oakville, ON, Canada) unless otherwise specified.

### 4.8. Data and Statistical Analysis

Contraction data were expressed relative to each vessel’s own sham control value. Statistical significance was then assessed on the raw data using ANOVA with an appropriate post-hoc test, as indicated in the text. Experimental data were expressed as mean values ± one standard error of the mean (SEM) and *p*-values less than 0.05 were considered significant.

## 5. Conclusions

We demonstrate for the first time that the lactone ring-containing prodrug forms, but not the hydrolyzed forms, of popular statins Lovastatin (Mevacor) and Simvastatin (Zocor) have deleterious impacts on lymphatic vascular function. This is shown to primarily affect the lymphatic smooth muscle cells, as hypothesized, through disruption of their mitochondrial network (Simvastatin) or regulation of contractile protein phosphorylation (Lovastatin). Critically, Lovastatin-induced mesenteric lymphatic contractile disfunction provides an explanation for the impeded intestinal lipid absorption and dissemination we observed in vivo. Therefore, these data suggest a novel mechanism by which statins act to reduce systemic cholesterol levels by reducing dietary lipid uptake. It will be important to consider our data and any underlying lymphatic comorbidities when assessing patient responses to statin therapy, as exacerbation of lymphatic disfunction, and consequent lymph stasis, may have stark pathological consequences.

## Data Availability

The data that support the findings of this study are available from the corresponding author upon reasonable request.

## Supplementary Materials

The following are available online at www.mdpi.com/article/10.3390/ijms222111756/s1.
