# Supplementary material for: Off-Target Effect of Lovastatin Disrupts Dietary Lipid Uptake and Dissemination through Pro-Drug Inhibition of the Mesenteric Lymphatic Smooth Muscle Cell Contractile Apparatus
Source: Int J Mol Sci. 2021 Oct 29;22(21):11756. doi: 10.3390/ijms222111756 (PMC8584239; doi:10.3390/ijms222111756)
Supplement: Supplementary file 1 [file ijms-22-11756-s001.zip › ijms-1413659-supplementary.pdf]

Supplemental Material

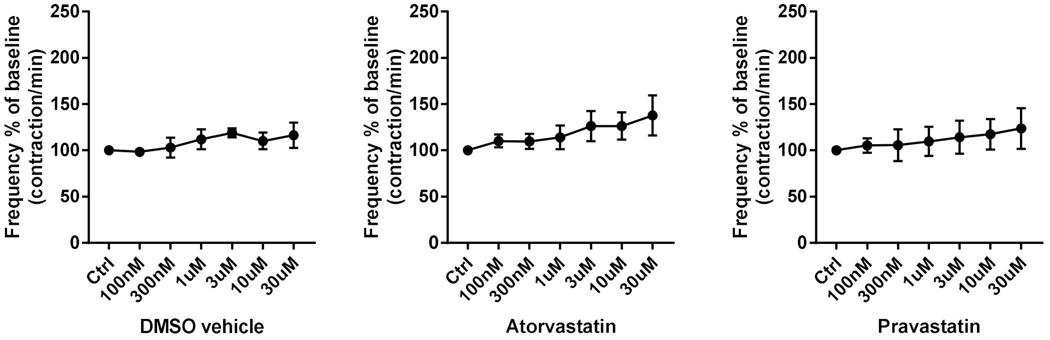

Supplemental Figure S1: Concentration-dependent contraction frequency curves. Isolated and pressurised rat mesenteric lymphatic vessels were incubated with increasing concentrations of the statins or control DMSO (**A-C**) Contraction frequency of vessel expressed as baseline corrected percentage for each vessel tested. Vessel contraction frequency is represented as mean ± SEM of n=5-12 individual experiments. *P<0.05, ***P<0.001, ****P<0.0001 vs baseline. One-way Anova with Dunnet's multiple comparisons test.
